# Applying the iDARE Methodology in Uganda, Kenya, and Tanzania to Improve Health Outcomes During the COVID-19 Pandemic

**DOI:** 10.9745/GHSP-D-21-00623

**Published:** 2022-06-29

**Authors:** Amanda Ottosson, Joyce Draru, Luseka Mwanzi, Stella Kasindi Mwita, Sara Pappa, Krista Odom, Taroub Harb Faramand

**Affiliations:** a WI-HER, Stockholm, Sweden.; b WI-HER, Mbale, Uganda.; c WI-HER, Nakuru, Kenya.; d WI-HER, Dar es Salaam, Tanzania.; e WI-HER, Vienna, VA, USA.; f WI-HER, Addis Ababa, Ethiopia.

## Abstract

**Introduction:**

We introduce the iDARE methodology and present the results of iDARE implementation in Uganda, Kenya, and Tanzania during the coronavirus disease (COVID-19) pandemic.

**iDARE Methodology:**

iDARE drives locally led solutions that address barriers to achieving improved health outcomes. WI-HER supported the governments of Uganda, Kenya, and Tanzania, to design and implement solutions to improve (1) HIV health outcomes, (2) gender-based violence identification and response, and (3) mass drug administration coverage, respectively.

**Results:**

In Uganda, the iDARE team at Nagongera Health Center IV increased viral load suppression (VLS) among actively enrolled men in care from 65% to 95% and increased VLS among actively enrolled children in care from 60% to 96% in 12 months. In 11 months, the Mulanda Health Center IV iDARE team increased VLS among actively enrolled men in care from 85% to 93% and actively enrolled children in care from 73% to 96%. In Kenya, 8 facility iDARE teams improved identification, management, and response for gender-based violence survivors by a monthly average of 642% in 10 months. Additionally, the identification, management, and response for male survivors of gender-based violence increased from an average of 8 to 188 men per month and from an average of 81 to 364 women per month. In Tanzania, the government applied iDARE to improve mass drug administration (MDA) access and uptake among school-age children. Eighteen percent of the children (equal male and female) had missed or refused treatment during school-based MDA. After 1 month of application of iDARE, the 4 schools achieved 99% MDA uptake among registered children (enrolled and nonenrolled).

**Discussion:**

Due to the various lockdowns, restrictions, and safety implications during the COVID-19 pandemic, iDARE was used to rapidly adjust from planned in-person to sometimes virtual engagements. Despite these challenges, iDARE demonstrated improvements in Uganda, Kenya, and Tanzania.

## Abstract (precis)

The iDARE methodology was implemented in Uganda, Kenya, and Tanzania during the COVID-19 pandemic to help build the capacity of local governments, facilities, and communities to identify, design, and implement local solutions to health problems. These solutions can be adapted and applied in any context with low-cost implications.

## Teaser

**Key Findings:**

- The iDARE methodology promotes locally led transformation that produces long-lasting, community-based change.
- During the ongoing COVID-19 pandemic, individuals and communities need to be met with cultural humility and equipped with innovative, participatory tools that enable them to design and achieve meaningful behavior change to improve outcomes.
- iDARE was rapidly and sustainably institutionalized in 3 country contexts with different targeted health outcomes, and improvement occurred in all outcomes.

**Key Implications:**

- Program managers (including civil society organizations, implementing partners, governments, and donors) may use the iDARE methodology for guidance on critical thinking on locally identified issues and developed solutions, leveraging existing resources, regularly monitoring progress, navigating challenges, and adhering to a “do no harm” principle, especially during the COVID-19 pandemic.

## INTRODUCTION

Understanding the influence of social norms and how they perpetuate harmful or restrictive gender-related perceptions and expectations that govern the individuals' ability to engage in health-seeking behaviors has become a priority. Norms are held in place through positive or negative sanctions, causing people to conform to group expectations out of the human need for social approval and belonging.^1^ As such, measuring and changing social norms, thus allowing new behaviors to emerge, may be more effective than attempting to modify individual attitudes and beliefs alone. Finally, capturing false consensus is important because rectifying false perceptions can be an important tool for behavior change.^2^

Consensus is still limited on how to adequately and effectively measure social and gender norms at scale, especially as they relate to specific health-seeking behaviors and social and behavior change (SBC) at large. As SBC can be difficult to measure and is prone to biases—because of self-report measures or social desirability bias, for example—proxy measures, such as intention to carry out a certain behavior or even positive attitudinal changes, are often used in assessments. In addition, behavior change is prone to fade-out effects.

To innovatively bridge the gap between implementation and behavior change sciences, WI-HER's president and founder, Dr. Taroub Harb Faramand, designed the iDARE methodology to innovatively bridge the gap between implementation and behavior change sciences. With gender equity and social inclusion (GESI) at its core, iDARE is used to build capacity, drive locally designed and led solutions, and ensure data use and accountability.^3^

The iDARE methodology was designed to innovatively bridge the gap between implementation and behavior change sciences.

In this article, we introduce the iDARE methodology and describe its application in Uganda, Kenya, and Tanzania, respectively, to improve (1) HIV health outcomes, (2) gender-based violence (GBV) identification and response, and (3) mass drug administration (MDA) coverage for neglected tropical diseases (NTDs), respectively, during the coronavirus disease (COVID-19) pandemic. We describe how certain aspects were adapted because of the pandemic to ensure safety and adherence to various country restrictions so that service delivery and overall health outcomes could still be improved and how iDARE worked at the implementation level by local stakeholders with WI-HER technical support.

## IDARE METHODOLOGY

**FIGURE 1:**
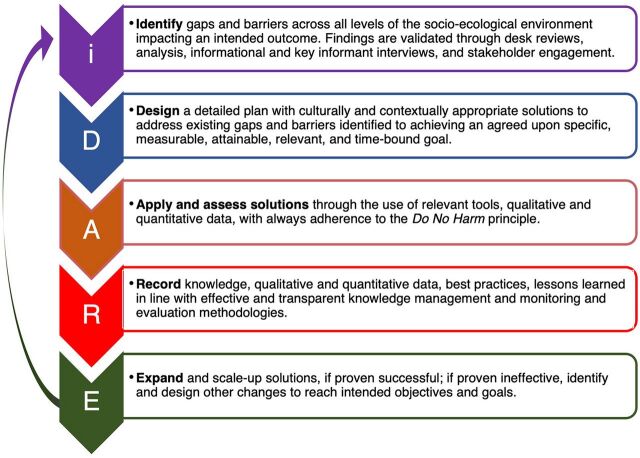
iDARE Methodology

The iDARE methodology (Figure 1) applies improvement science to drive locally led solutions. The methodology is grounded in human-centered design and draws from classic behavior theories and concepts that have a robust evidence base and application in the SBC field, such as the theory of planned behavior,^4^ social cognitive theory,^5^ and diffusion for innovations,^6^ as well as innovative approaches from parallel fields including behavioral science, behavioral economics, and consumer/marketing research.

iDARE facilitates a deeper understanding of intersecting sociocultural, behavioral, and contextual factors as they relate to desired outcomes. The methodology is rooted in the fundamental principle that addressing persistent inequities requires starting where people are^7^ and putting people at the heart and in the driver's seat of solutions. iDARE incorporates empowerment as an action-oriented concept with a focus on communities achieving equity by removing barriers and on transformation of power relations among individuals, communities, and institutions, including those related to gender and cultural norms surrounding gender.^8^^–^^10^ Thus, rather than an externally imposed action, it is a participatory, enabling process through which individuals and/or communities express agency and take control of their lives and environments.^11^ iDARE catalyzes locally led community program design, implementation, monitoring, evaluation, and learning.

Similar to the plan-do-study-act (PDSA) change model originating from the “articulation of iterative processes,”^12^ iDARE is a 5-step, iterative process that allows for frequent, rapid assessment, learning, and adaptation of ongoing work. However, iDARE goes beyond the PDSA change model. Throughout all stages of iDARE, particular attention is paid to investigating and understanding power dynamics and influencing factors—including individuals, institutions, environment, social, and cultural factors—in the formal and informal local systems, within the context of the existing community system. Importantly, gender is foundational in the understanding of power dynamics and cultural factors that is the lens through which iDARE is implemented. These additional socioenvironmental considerations have proven critical in improving service delivery because the methodology goes beyond focusing on improvement processes, including in gender. It factors in the human experience, including sensitively identifying and addressing bias, stigma, discrimination, perceptions, attitudes, and beliefs of all stakeholders that play a role in the socioecological environment of the work at the policy and legal level.

At the program implementation level, iDARE teams receive coaching support on using the methodology, thinking through the issues, identifying problems to address, and then designing culturally and contextually appropriate solutions that are locally led and sustainable. Solutions are designed to leverage existing resources, including but not limited to human resources, financial (donor, community, and/or host country government), partnerships, and ongoing development efforts, as well as working within the existing system and structures of the socioecological environment. This approach means that other government and/or donor-funded projects can be leveraged to achieve an iDARE team's goal. iDARE application is informed by behavioral tools and levers, and it is always adapted to ensure that the teams incorporate knowledge of culturally specific psychological biases and bottlenecks. This is done throughout implementation when tools are adapted with the local stakeholders to address the specific needs of the context.

## iDARE IMPLEMENTATION IN UGANDA, KENYA, AND TANZANIA

In 2020, through 3 separate projects funded by the U.S. Agency for International Development (USAID) that focused on improving social and behavior change, improving HIV services, and eliminating NTDs, WI-HER began to support the governments of Uganda, Kenya, and Tanzania, respectively, to design and implement locally led solutions to achieve the desired outcomes. These countries were not selected specifically to implement iDARE but because of WI-HER's role as a subcontractor focusing on integrating GESI across project activities as well as identifying and addressing GESI barriers affecting project goals.

In all countries, the original programming intention was to offer in-person support to local governments to achieve their goals over a longer period than what took place. However, given the global COVID-19 pandemic, the Ugandan, Kenyan, and Tanzanian governments, as well as the respective USAID missions, implemented disease control mitigation strategies to ensure the safety of country residents and citizens. The public health measures, including lockdowns and restrictions on movement, caused significant delays in implementation and resulted in innovative adaptions, moving from in-person plans to new operating environments. While the pandemic heavily affected health workers and the systems at large, services needed to continue. iDARE provided an opportunity to build local capacity to navigate challenges and identify priorities to address based on emerging needs. Restrictions varied heavily across the 3 countries and occurred during different periods.

Amid COVID-19-related difficulties, iDARE provided an opportunity to build local capacity to navigate challenges and identify priorities to address based on emerging needs.

The iDARE methodology was applied at various levels by local stakeholders with coaching support. iDARE coaching traditionally takes place in person, with teams convening to meet the iDARE team coach, trained in the methodology under Dr. Faramand, and review progress to date using evidence gained; assess if solutions tested were successful and should be scaled up/continued, or if they were instead non-successful; identify new gaps, barriers, and/or issues for achieving the intended outcome; and design new solutions to test. However, because of the pandemic, coaching was virtual (Zoom), via telephone, in person with small groups, and/or a hybrid approach (mixed virtual and in person). Teams were guided by the coach on regular monitoring of data and retiring activities that no longer met a minimum threshold of viability. Furthermore, after iDARE teams started to achieve positive results and became independent, the team coach supported them in identifying opportunities in expanding iDARE work to a new goal or, in the case of behavior change, potentially reach a new subgroup or population. We present 3 parallel case studies of iDARE implementation from Uganda, Kenya, and Tanzania.

## IMPROVING HIV HEALTH OUTCOMES IN UGANDA

### Step 1: Identify Gaps in Health Outcomes

iDARE implementation in Uganda began in August 2020 when WI-HER, through the USAID Social and Behavior Change Activity (2020–2025), met with the Tororo District Local Government (DLG) and the regional implementing partner to review district-level data (DHIS2) to identify an area to begin work in improving health outcomes through behavior change interventions. DHIS2 showed that HIV-related health outcomes were the furthest from achieving their targets, with men (aged 20 years and older) and children (aged 19 years and younger) having the largest gap, specifically with the third HIV indicator, viral load suppression (VLS). The DLG reviewed the data and identified a facility to begin work. Table 1 shows Nagongera Health Center IV's (HCIV) clients who were virally suppressed (of those enrolled and active in care) at the time of the baseline. In September 2020, the iDARE coach supported the DLG to repeat this process and identify a second facility, Mulanda HCIV (Table 2), to begin work and support learning within the district among the 2 catchment areas. In contrast to Nagongera HCIV, Mulanda HCIV had a higher percentage of virally suppressed men and children active in care, while women had a lower percentage (84% suppressed) compared with 92% of Nagongera's actively enrolled adult female clients. Moreover, facility-level data revealed that in addition to VLS being an area that needed improvement for all clients, the total numbers of men and children active in care were low. It is critical to note that the iDARE teams were focused on behavior change of individuals in care and not service delivery improvement. Services continued to be regularly provided at the facility level throughout the iDARE implementation.

**TABLE 1:** Nagongera Health Center IV, Uganda, iDARE Baseline Assessment of Clients Virally Suppressed, August 2020

	Total Clients Active in Care	Total Suppressed, No. (%)
Women (age 20 years and older)	883	811 (92)
Men (age 20 years and older)	550	357 (65)
Children (age 19 years and younger)	103	62 (60)
Girls	56	40 (71)
Boys	47	22 (47)

**TABLE 2:** Mulanda Health Center IV, Uganda, iDARE Baseline Assessment of Clients Virally Suppressed, September 2020

	Total Clients Active in Care	Total Suppressed, No. (%)
Women (age 20 years and older)	637	536 (84)
Men (age 20 years and older)	347	296 (85)
Children (age 19 years and younger)	52	38 (73)
Girls	29	21 (72)
Boys	23	17 (74)

iDARE teams in Uganda focused on behavior change of individuals in care, not service delivery improvement.

With sustainability and institutionalization planned at the project onset, initial iDARE teams were formed from existing personnel with key individuals identified and recommended by the appropriate local government structures, the DLG and the health facility's in-charge. Then, the teams recruited additional members from the community. Community influencers were selected through informal interviews with the target populations or a cohort (small group of nonsuppressed clients) to ensure the team(s) and overall process were community driven. The initial Nagongera HCIV iDARE team established a consenting cohort of 14 nonsuppressed men and 16 nonsuppressed children from the community who were actively enrolled in care. Mulanda HCIV followed suit, establishing an initial cohort of 14 men and 12 children. Both teams then met with the men, children (when they were over assenting age), and caretakers to understand gender, youth, and social inclusion root causes that may be linked to their behavior. For children, lack of adult support was identified as a barrier to antiretroviral therapy continuity and therefore, low levels of VLS. For men, work and timing for clinic visits led to low levels of antiretroviral therapy continuity and therefore low levels of VLS. In these informal interviews, the iDARE team sought to understand who influences cohort members' beliefs, attitudes, perceptions, opinions, and decisions that lead to a behavior. After identifying influencers mentioned by the various cohort members, the iDARE team recruited the influencers to join the team.

### Step 2: Design Solutions to Increase VLS

The iDARE team coach supported Nagongera HCIV and Mulanda HCIV iDARE teams to analyze the findings from interviews with the cohort members to identify root causes of the behavior of the men, children, and the children's caretakers; document all root causes and prioritize issues to address; and brainstorm and design solutions to test and assigned individuals from the team to lead the implementation of the solution. Team leaders followed up with the responsible team members to check progress on a regular, agreed-upon basis.

### Step 3: Apply Solutions and Assess Progress

iDARE teams in both facilities met to assess their progress on the agreed-upon solutions, review facility data, continue coaching through the iDARE process, and hold learning sessions. The iDARE team coach, along with iDARE team supervisors in local government structures, provided formal coaching sessions at least once a month. Because of COVID-19 regulations and safety measures, coaching was conducted virtually, in person where possible, and through a hybrid approach of virtual and in person.

The iDARE coach also conducted multiple in-person and virtual learning sessions for teams from Nagongera HCIV and Mulanda HCIV that allowed the facility teams to reflect and learn from each other on what worked and what did not and to stimulate new ideas that addressed common issues.

### Step 4: Record Results and Document Lessons Learned

Table 3 shows successful and nonsuccessful solutions applied during Nagongera HCIV's and Mulanda HCIV's iDARE implementations. Learnings were harvested and shared between the 2 facilities at a learning session, which led Mulanda HCIV to test a Nagongera HCIV successful solution to address the lack of food for children for non-suppressed child clients at Mulanda HCIV. The iDARE team from Mulanda HCIV determined the solution was not a success because the religious leader was not as active in the community as the Nagongera pastor and was unable to mobilize food as easily. The Mulanda HCIV team further brainstormed to understand why the tested solution did not succeed as it did in Nagongera. First, the recruitment of the religious leader to the Mulanda HCIV team was done differently from that conducted by the Nagongera HCIV's team. In Nagongera, the pastor was identified as an influencer during the informal interviews with the 16 nonsuppressed children and their caretakers and asked to join the iDARE team from the beginning of implementation. In contrast, Mulanda HCIV specifically sought to find a religious leader as an influencer. They spoke to 3 caretakers of nonsuppressed actively enrolled child clients separately and asked who the influential religious leader was in the community; all named the same person. Health workers at the facility also identified the same individual when asked the same question. No children (active in care) or additional caretakers, beyond the previously mentioned 3, were consulted. The religious leader identified was asked to join the iDARE team and implement the successful solution from Nagongera; however, the religious leader from Mulanda was not as motivated nor as active as the pastor from Nagongera. This outcome serves as an example of how a solution must be appropriately adapted to the context and not duplicated. A potential way to adapt the solution in Mulanda could have been to ask 1 or more community influencers—identified by nonsuppressed children and where applicable, their caretakers—to lead the solution of mobilizing food within the community at a set time during the week.

**TABLE 3:** Sample Issues and Solutions Applied During iDARE Implementation to Improve Viral Load Suppression Among Children Active in Care, Nagongera Health Center and Mulanda Health Center, Uganda

Issue	Solution Tested	Facility	Successful/Not Successful
Lack of support from parents	Case-by-case basis: One community linkage facilitator and the pastor (influencers) encourage parent(s) to bring children to the facility for their appointments, ensure they have food to take with medication (see below), and provide transport (if they are unable to come, they send the child with a responsible person or organize transport). See children and adolescents on Thursdays at the facility.	Nagongera	Successful
Staying with elderly (such as grandparents)	Community linkage facilitator (also the identified community influencer on iDARE team) takes medication to child at home; pastor (influencer) also conducts home visits with these children. Pastor organizes transport (when needed) to the facility for their appointments.	Nagongera	Successful
Lack of food	Religious leader, identified in both areas as a community influencer and recruited for iDARE team, designated to lead support for the children. The religious leader has done outreach and lobbies community members during designated Sunday church services to contribute food for the children.	Nagongera	Successful
		Mulanda	Not successful

The outcome in Mulanda serves as an example of how a successful solution in one context must be appropriately adapted to another context and not just be duplicated.

### Step 5: Expand Efforts to More Clients

The 2 facility iDARE teams in Tororo District expanded their efforts in multiple ways. In Nagongera HCIV: (1) after 2 months, the team expanded beyond their initial cohort of 30 nonsuppressed men and children active in care to further identify, design, and apply solutions for all nonsuppressed men and children (Table 3) who were actively enrolled in care; and (2) after 3 months, began to work on lost to follow-up among men.

In Mulanda HCIV, the team: (1) after 3 months, expanded their efforts to work on lost to follow-up among children; (2) after 5 and 9 months, respectively, expanded to improve VLS among active men and children by establishing 2 additional cohorts; and (3) after 6 months, with support from the DLG, identified TB case notification as another priority for iDARE application.

### Results: Increased VLS Among People in Care

**FIGURE 2:**
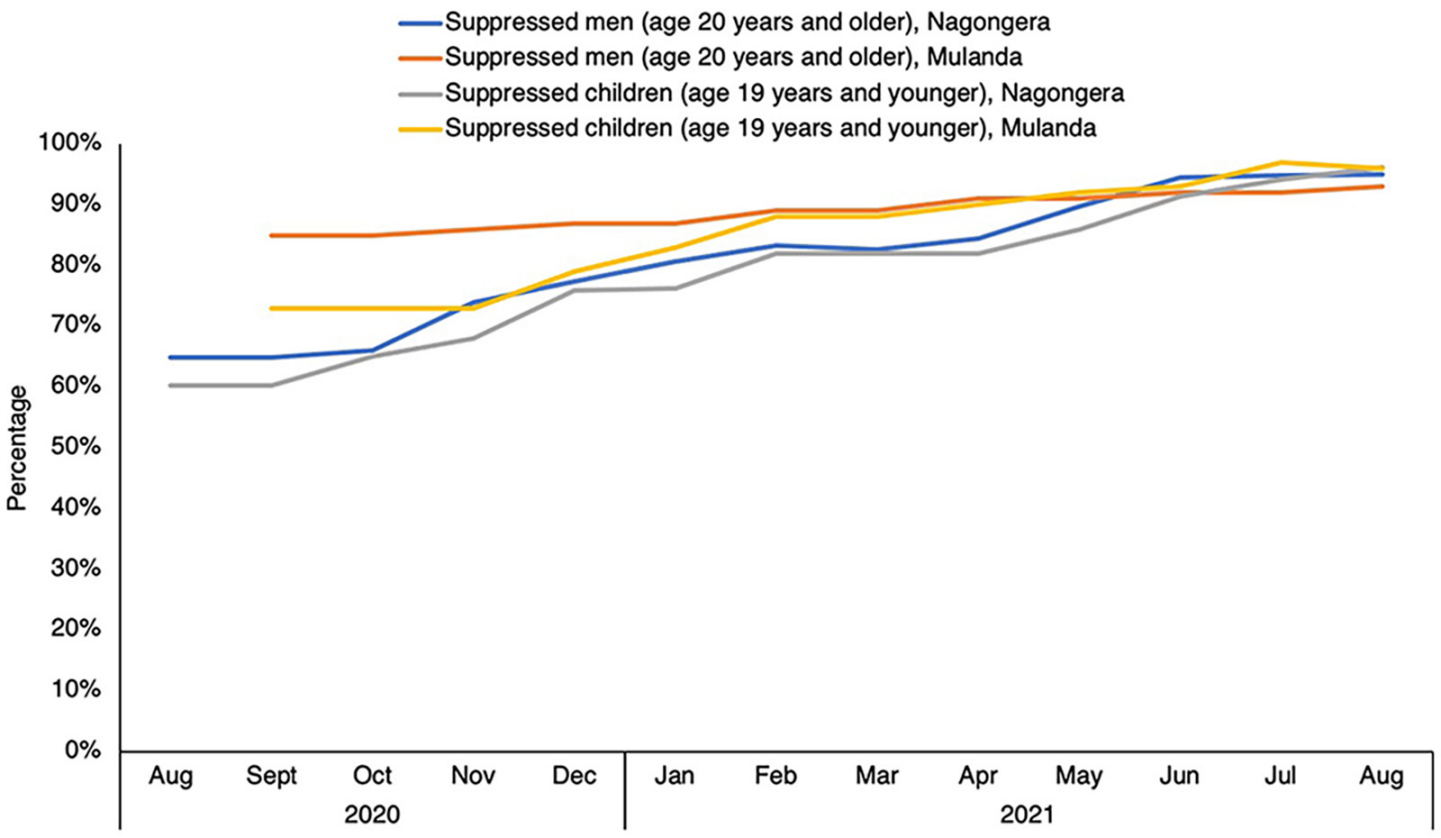
Percentage of Enrolled Men and Children Active in Care Who Were Virally Suppressed During iDARE Implementation, Nagongera Health Center and Mulanda Health Center, Tororo District, Uganda

In 12 months, the iDARE team at Nagongera HCIV increased VLS among actively enrolled men in care from 65% (357/550) to 95% (534/562) and increased VLS among actively enrolled children in care from 60% (62/103) to 96% (100/104). Furthermore, they closed the gender gap among male and female children from 47% (22/47) and 71% (40/56), respectively, to 94% (48/51) of male children suppressed and 98% (52/53) of female children suppressed. In 11 months, Mulanda HCIV's iDARE team increased VLS from 85% (296/347) to 93% (324/350) among actively enrolled men in care and from 73% (38/52) to 96% (75/78) among actively enrolled children in care. A gender gap did not exist among children at Mulanda HCIV at baseline, and the iDARE team ensured that a gap did not emerge during the 11-month period, achieving 97% (36/37) male and 95% (39/41) female children suppressed from 74% (17/23) and 72% (21/29), respectively (Figure 2).

## IMPROVING IDENTIFICATION AND MANAGEMENT FOR SURVIVORS OF GBV IN KENYA

### Step 1: Identify Gaps in GBV Identification and Management

In May 2020, WI-HER, through the USAID Afya Nyota ya Bonde project (2018–2021), used program data to identify facilities with the lowest identification of, management of, and response for GBV survivors. All facilities supported by the Afya Nyota ya Bonde project were given GBV identification and management targets estimated by USAID and the U.S. President's Emergency Plan for AIDS Relief (PEPFAR) based on the catchment area and the number of clients and facility size. GBV refers to harmful acts directed against an individual based on their gender.^13^ Although it disproportionately affects women and girls, GBV can involve individuals of both sexes and all genders. In Kenya, survivors are classified by sex, male and female. A GBV survivor is any male or female who has been or is being subjected to 1 or more of the 4 forms of GBV, namely, emotional, physical, sexual, and financial.^13^ The WI-HER team identified 8 facilities in 3 counties, Nakuru, Laikipia, and Kajiado, in Kenya with the estimated highest burden of GBV survivors, which accounted for 59.7% of the entire GBV PEPFAR target for all 419 project-supported facilities. After analysis of program data, the iDARE coach worked with 3 (of 5 project-supported) county government teams to identify up to 4 health workers per facility for training on all forms of GBV, World Health Organization (WHO)'s LIVES,^14^ and iDARE methodology. Before the training, these health workers submitted their facility-level baseline performance data from January to May 2020.

At baseline, on average, the 8 facilities had achieved a 4% average of their estimated facility-level PEPFAR targets, which is an aggregated target for males and females of all ages, including children. Furthermore, facilities and health workers only had the capacity and tools for the identification of, management of, and response for survivors of sexual violence. Health workers were not equipped with the knowledge, skills, or tools to identify, provide care, and/or report for survivors of emotional, financial, or physical violence. Additionally, across all facilities, there was extremely low identification and management of male survivors of violence, with an average of only 8 male survivors identified per month (Table 4).

**TABLE 4:** Monthly Number of Survivors of Violence (All Ages) Identified and Managed in 8 Facilities During Baseline by iDARE Team, 3 Counties, Kenya, 2020

	January	February	March	April	May
Monthly PEPFAR target	2,494				
Female survivors	79	71	89	70	95
Male survivors	9	4	6	12	7
Percentage of monthly (male and female) PEPFAR target, %	4	3	4	3	4

Abbreviation:; PEPFAR, U.S. President's Emergency Plan for AIDS Relief.

WI-HER adapted existing Ministry of Health tools, approaches, and standard operating procedures to meet international standards and trained health workers on their usage. Furthermore, the training had extensive practical sessions on stigma and bias in GBV care and response. Health workers immediately began testing the tools in their facilities and provided feedback in real time to the iDARE team coach to ensure tools were practical and contextually relevant. Additionally, to ensure and measure GBV quality of care within facilities, WI-HER developed a coaching guide and GBV quality assurance tool to assess gaps in the provision of the minimum package of care for survivors of GBV. The GBV quality assurance tool uses direct observation; inquiry with providers and facility managers; and review of clinical records, guidelines, protocols, and documents to assess facilities based on the following domains: availability and appropriateness of services, facility readiness and infrastructure, case findings of survivors of GBV, survivor-centered clinical care and provider-survivor communications, forensic examination and handling of evidence, referral system and follow-up with survivors, reporting and information systems, training and quality improvement, and health care policy and provision.

Initial iDARE teams in Kenya were formed with the county government GBV focal person and health facility in-charge identified and recommended by the appropriate local government structures and health workers at the facilities.

iDARE work in facilities focused on service delivery improvement. The initial iDARE team also included health workers who served the 3 critical functions of the continuum of care for GBV survivors: (1) identify and screen cases (generally stationed in the outpatient department); (2) provide GBV counseling and psychosocial support; and (3) clinicians (nurse/doctor/clinical officer) who conduct full physical and, when appropriate, gynecological examinations. In most cases, a health worker served more than 1 of the functions. The virtual training was conducted over 6 weeks with low-dose, high-frequency training and coaching sessions. The health workers were trained on all 4 forms of GBV, expanded GBV tools, simplified WHO LIVES,^14^ and iDARE. The trained health workers then identified additional team members within their facility to support their iDARE work. Most facilities recruited at least the health information officer as well as representatives from other departments to improve identification throughout the facility.

### Step 2: Design Solutions to Strengthen Capacity in GBV Identification and Management

Initial iDARE teams designed early solutions to test, along with plans to recruit additional members to the iDARE team, during their 6-week virtual training. As the teams expanded, they designed additional solutions to address gaps they identified with colleagues from other departments in their facilities. Furthermore, the iDARE team coach engaged county government officials to support the institutionalization of the work to improve GBV identification and management in the 3 counties. This resulted in 1 county identifying and establishing a GBV focal person for a facility that did not have one.

### Step 3: Apply and Assess Solutions Tested

The iDARE team met regularly to assess facility progress on the agreed-upon solutions, review facility-level data (e.g., the GBV register), continue coaching through the iDARE process, and hold learning sessions. WI-HER, along with iDARE team supervisors in local government structures, provided formal coaching sessions at least once a month. Coaching was conducted virtually, in person where possible, and through a hybrid approach of virtual and in person due to COVID-19 regulations and safety measures that were in place.

Strengthening capacity ranged from reorienting teams and new team members on the expanded GBV tools, including the GBV checklist and quality assurance tool, or conducting training on the forms of GBV and how to identify, manage, and respond to a survivor based on the minimum package of care. The iDARE team coach supported the teams to identify and record additional gaps and barriers they experienced, brainstorm new solutions to begin testing, and supported the teams in the quantitative and qualitative data recording and analysis.

Due to the distance between facilities and COVID-19 movement restrictions during lockdown, learning sessions took place over Zoom. While a learning session has the greatest impact when all team members can participate, in Kenya the facilities were heavily affected by COVID-19 and could not afford to have multiple team members away from their stations even for 1- or 2-hour sessions. Therefore, Kenya teams suggested having only their iDARE team members participate in learning sessions and report back to their wider team. Although recordings of the learning sessions were shared with all team members, the use of Zoom was less interactive and engaging than in-person learning sessions. Data and network challenges caused issues in sound and overall participation. It was also difficult for the health workers who could not attend to find time to download and review the recordings to hear the learnings from other facilities.

### Step 4: Record Successful Solutions in Improving GBV Identification and Management

Table 5 shows identified gaps in GBV care and the tested successful sample solutions recorded in a provincial general hospital–county referral in Kenya that was not an early adopter of the iDARE methodology or improving GBV identification and management. However, once the hospital began to see early results showing improvement, it became more confident in implementing iDARE and greatly improved GBV identification and management of female and especially male survivors.

**TABLE 5:** Gaps and Successful Sample Solutions Applied In Identifying and Managing Survivors of Gender-Based Violence, Recorded by Provincial General Hospital County Referral iDARE Team, Kenya

Gaps in GBV Identification and Management	Successful Solutions
Lack of identification points	Sensitize management and other departments, including training on screening and referral of survivors to the GBV clinic.
Lack of provider knowledge and skill to care for GBV survivors	
Reporting tools only identify sexual GBV	Implement new registry that reports on physical, emotional, financial, and sexual violence.
Single GBV registry for case reporting, and no registry at the drop-in center	Provide expanded GBV registry for each department.
	Provide expanded GBV registry for the drop-in center.
Staff shortage and high workload, especially during COVID-19 pandemic	Sensitize and train community health volunteers and community health workers on GBV care.
Lack of community knowledge of resources	Provide health talks to the community, targeting survivors.
	Provide literature to survivors with information on services.
Disorganized GBV data management across departments	Form a WhatsApp group to assist in the management of data.

Abbreviations: COVID-19, coronavirus disease; GBV, gender-based violence.

### Step 5: Expand Awareness of Availability of GBV Care

iDARE teams worked on expanding their efforts in the community to ensure that individuals who have been subjected to violence know there are support services available to them at the facility (Table 5).

### Results: Increased GBV Identification and Management

**FIGURE 3:**
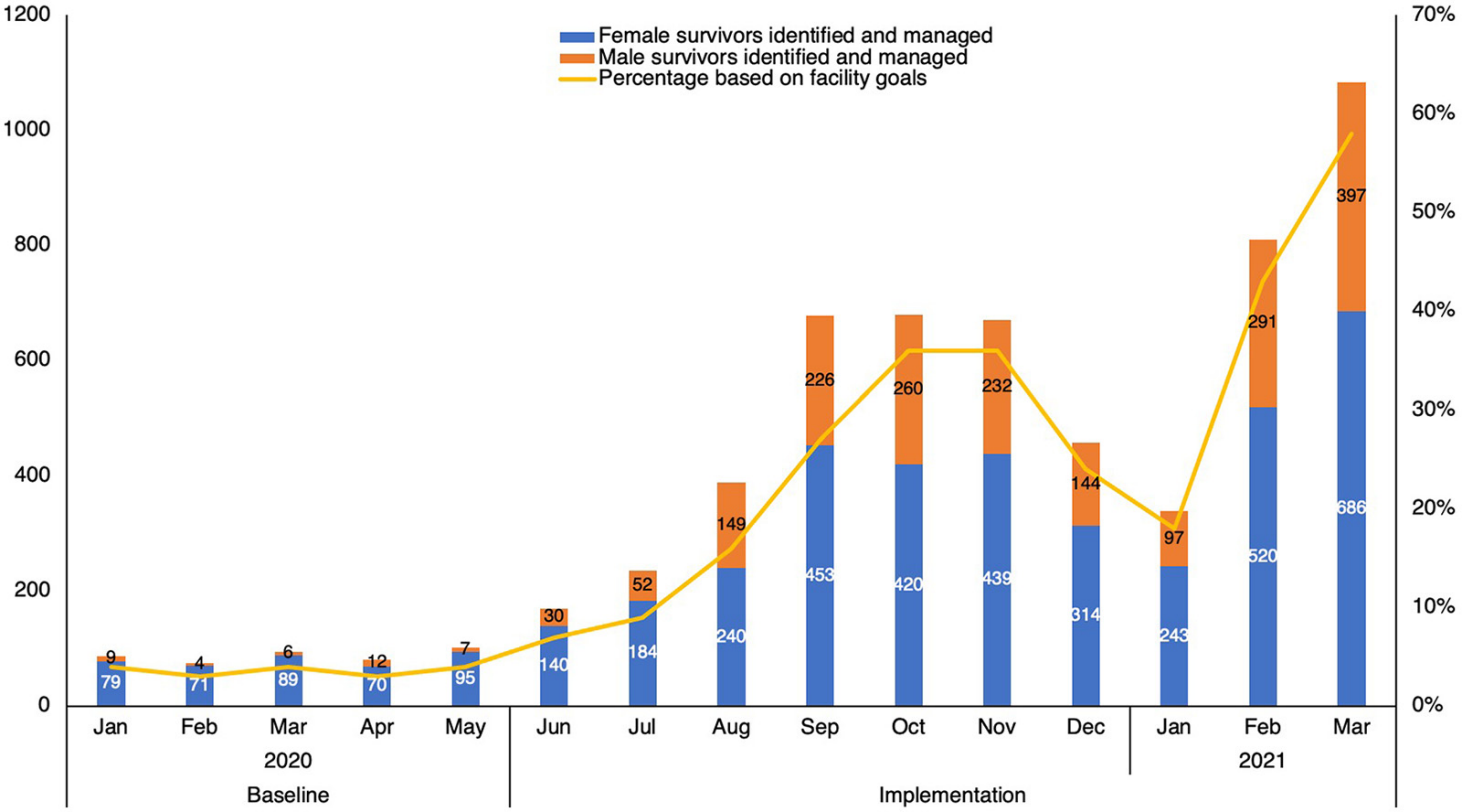
Gender-Based Violence Cases Identified and Managed by iDARE Teams, by Sex, in 8 Facilities in Nakuru, Laikipia, and Kajiado Counties, Kenya, January 2020–March 2021

In 10 months of implementation, the 8 facility-based iDARE teams increased GBV identification and response by 641.29% for male and female survivors of all 4 forms of violence. From June 2020 to March 2021, a total of 5,517 cases of GBV were identified, with an average of 551 persons per month. The 8 facilities increased identification and management of female survivors from an average of 81 per month (baseline) to 364 female survivors per month (June 2020 through March 2021). Additionally, the facilities increased identification of male survivors from an average of 8 survivors per month (baseline) to 188 male survivors per month (June 2020 through March 2021), resulting in a 2,250% increase in male survivors per month identified and managed. These results demonstrate the progress the facilities made, despite the COVID-19 pandemic and a health worker strike in December 2020 and January 2021 (Figure 3).

In 10 months of implementation, the 8 facility-based iDARE teams in Kenya increased GBV identification and response by 641.29% for male and female survivors.

## IMPROVING MDA COVERAGE FOR NTDS IN TANZANIA

### Step 1: Identify Gaps in Coverage of MDA

In January 2021, WI-HER, through the USAID Act to End Neglected Tropical Diseases—East Program (2018–2023), began supporting the Tanzania National NTD Control Program (TZNTDCP) to review national NTD program data, including surveys evaluating coverage of MDA for onchocerciasis and trachoma treatment to identify a district to begin testing iDARE to improve coverage for frequently missed or refusing populations. The national GESI assessment identified men who take alcohol and school-age children as the most commonly missed or refusing groups for community and school MDA, respectively.

In March 2021, in the Pangani district, the TZNTDCP worked with district council leadership to use existing data (Table 6) and conduct community-based informational interviews to identify GESI challenges for community and school MDA access and uptake of the deworming medication albendazole. To explore low access and uptake among both types of MDA, the implementation team sought to understand if those targeted during community MDA, specifically adults, influence their children's behavior during school MDA. Data were collected from the MDA registers by the district NTD teams during the pilot. From the above information, 13 schools in 2 zones—a group of wards, which constitute a group of villages—showed an average gap of 20% for both female and male school children in the available disaggregated data. Two schools in each of the Mwera and Mkwaja zones that had the largest absolute number of missed school-age children were selected for application of iDARE to improve access and uptake of MDA.

**TABLE 6.** Enrolled School Children Who Received MDA for NTDs and Those Who Missed in 13 Primary Schools in 2 Zones of Mwera and Mkwaja, Pangani District, Tanzania

Zone	Primary School	Target Population			Missed MDA		
		Male	Female	Total	Male, No. (%)	Female, No. (%)	Total, No. (%)
Mwera	Mwera^a^	384	408	792	28 (3.5)	79 (10.0)	107 (13.5)
	Mzambarauni	46	54	100	8	11	19
	Ushongo	137	131	268	27	19	46
	Stahabu^a^	N/A	N/A	450	53 (11.7)	29 (6.4)	82
							(−18.2)
	Mikinguni	78	90	168	8	15	23
	Mtonga	133	106	239	35	34	69
	Mtango	194	176	370	18	18	36
Mkwaja	Sange	141	119	260	19	11	30
	Makorora	113	107	220	4	3	7
	Mikocheni	115	87	202	8	3	11
	Mkwaja	141	131	272	2	8	10
	Mkalama^a^	591	575	1166	196 (16.8)	175 (15.0)	371 (31.8)
	Mbulizaga^a^	173	169	342	47 (13.7)	28 (8.2)	75 (21.9)
Total		2246	2153	4849	453 (20.1)	433 (20.1)	886 (18.3)

Abbreviations: MDA, mass drug administration; NTDs, neglected tropical diseases.

a School selected for application of iDARE implementation to improve access and uptake of MDA.

Cohorts were formed in the 2 zones and represented various groups to understand the reasons for missing the last MDA (community, school, or both). For community MDA, reasons included people were not at home during MDA because of the nature of their livelihood activities (e.g., migration for farming, pastoralism, and fishing); households were too far for the community drug distributors to reach; fear of side effects, especially when mixing with other drugs for chronic diseases such as hypertension and diabetes; or the common belief among both men and women that MDA medicines could cause impotency and/or infertility. Reasons children missed or refused school MDA included school absenteeism; culture and traditions of the community; lack of food, which caused side effects and then fear; parents not allowing their children to take medication; fear of infertility; general fear; and discomfort in taking medication.

### Step 2: Design Solutions to Address Root Cause Affecting Access and Uptake

Initial teams were formed with key individuals identified and recommended by the appropriate government structures, including the NTDCP focal person, regional NTD representative, and district stakeholders.

The iDARE work in Tanzania focused on SBC. The initial iDARE team interviewed children, caretakers, and teachers on the various general and gender, youth, and social inclusion root causes affecting behavior, as well as the influencers in their lives. The influencers were then asked to join the iDARE team to support the behavior change work. The iDARE teams continuously expanded action plans before and during implementation with various members supporting the iDARE work, including supervisors and iDARE team members. iDARE team members in Pangani Council documented the identified barriers, gaps, and issues affecting MDA access and uptake and their designed solutions as well as results. It was adapted to the local language to be used by all team members.

Similar to the process in Uganda, the work in Tanzania focused on SBC and addressing the root causes of missing or refusing MDA.

### Step 3: Apply and Assess

Although the implementation period was short in Tanzania, the iDARE teams still met regularly to assess their progress on the agreed-upon solutions through the review data (document their solutions, measure intention among cohorts, and collect final uptake data from the MDA register), and receive coaching support from local government and iDARE team coach.

The iDARE team coach, along with team supervisors in local government structures, provided in-person coaching sessions throughout implementation. COVID-19 delays caused the length of the planned implementation to be shortened from at least 3 months to only 1 month; coaching was then provided intensively throughout the entire duration and in person. COVID-19 restrictions and time constraints prohibited additional coaching visits in Tanzania.

### Step 4: Record Progress Toward Intent to Take Medication

The teams in the Pangani district determined a proxy indicator, intent to take medication, as a means to measure progress of the intended outcome: uptake during the upcoming school MDA. Because of limited implementation time before the school MDA, only 1 measure of intention was taken after the team began testing solutions (Table 7).

**TABLE 7.** Results of iDARE Team Assessment for Proxy Indicator of Intention to Take MDA

Primary School	Group Who Did Not Take Last MDA for Assessment	Intent to Take MDA
Mkalamo	Parents of school children	51/52
	School children	34/35
Mbulizaga	Parents of school children	22/22
	School children	17/17
Mwera	Parents of school children	17/17
	School children	28/30
Stahabu	Parents of children	13/13
	School children	26/26
Total assessed for intent to take MDA		208/212
Intend to take next MDA, %		98

Abbreviations: MDA, mass drug administration.

In addition to the above-mentioned groups, iDARE teams also assessed community members in Mkalamo (15) and Mwera (7) to gain further insight into the communities' outlooks on MDA. These individuals were selected based on if they did not take the medication during the prior MDA. Twelve of 15 community members in Mkalamo and 7 of 7 community members in Mwera stated they intended to take medication at the next MDA. Additionally, as nonenrolled school children previously could not be verified by school MDA registers regarding whether they had taken medication, the iDARE team intentionally identified a group of nonenrolled children in Mkalamo to understand their intent to take the next MDA. All 24 of the nonenrolled school-age children intended to take the medication.

### Step 5: Expand to Additional Districts

After the successful improvement of school MDA coverage, the NTDCP held a reflection meeting on learnings from the Pangani District pilot and began plans to scale up to 6 additional districts to improve community and school MDA access and uptake for onchocerciasis and trachoma treatment.

### Results: Improved School-based MDA Coverage

After 1 month of implementing iDARE, the Pangani district achieved 99% coverage in the 4 pilot schools (Table 8). Of those nontreated at Mwera Primary School, 4 registered children had transferred to another school, 3 children were sick during MDA, and 20 were absent from school. In Stahabu Primary School, 1 child who was registered transferred to another school before MDA, and 1 child was pregnant and not offered the medication. iDARE teams have reviewed these reasons for missing MDA and have begun to design new solutions to improve the next MDA when the time comes.

**TABLE 8.** Results From 4 Pilot Schools Utilizing iDARE to Improve School MDA Coverage

Primary School	No. Enrolled	No. Not Enrolled	Previously Missed MDA, %	No. Children Treated (March MDA)		Not Treated March 2021 MDA	March 2021 MDA Coverage, %
				Enrolled	Not Enrolled		
Mkalama	1,185	39	32	1,185	39	0	100
Mbulizaga	366	0	22	366	0	0	100
Mwera	848	0	14	821	0	27	97
Stahabu	452	0	18	450	0	2	100

Abbreviations: MDA, mass drug administration.

After a single month of application of iDARE, the district achieved 99% coverage in the 4 pilot schools.

## DISCUSSION

While the evidence base for quality improvement and improvement science has increased, especially from low- and middle-income countries, much of the evidence informing SBC approaches comes from applications in high-income countries. Additionally, evidence from the COVID-19 pandemic is also limited and still emerging. The ramifications of the current COVID-19 crisis and associated factors—such as confinement measures, social isolation, financial stress, and weak institutional responses—have exacerbated harmful social and gender norms and jeopardized public health gains. Individuals and communities need to be met with cultural humility and equipped with innovative, participatory tools that enable them to design and achieve meaningful behavior change to improve outcomes. The highly adaptable iDARE methodology can provide this framework to donors, implementers, governments, and communities themselves to identify bottlenecks and barriers to achieving an intended outcome and design culturally and contextually appropriate solutions to complex social and system issues.^3^

From a sustainability perspective, iDARE builds the capacity of local governments, facilities, and communities to devise and implement local solutions to local problems; critically, these solutions can then be adapted and applied in any context with low-cost implications. Once individuals have received training on iDARE and coaching support, they can continue application beyond the project and/or intervention period as they can integrate application of iDARE into their daily work. In the case of Uganda, Kenya, and Tanzania, this application included national, regional, and local government stakeholders, health workers, community health workers, and community influencers. While the goals, health areas, and contexts largely varied in Uganda, Kenya, and Tanzania, the core application of iDARE remained the same throughout the 3 countries, with adaptations made for cultural and contextual differences (Table 9). All iDARE teams were given a package of highly participatory implementation tools, which were adapted and codeveloped with the teams to ensure contextual appropriateness and need: (1) the iDARE journal allows regular tracking of identified barriers and designed and implemented solutions, as well as progress using an agreed-upon measurement; (2) an iDARE guide that supports them in identifying barriers experienced by the target population, including GESI barriers and key influencing factors affecting individuals' lives; and (3) a root cause analysis tool that allows teams to interpret and analyze information they have received from the target population and begin to prioritize gaps and barriers they would like to begin to close. In some cases, additional tools can be developed to support the teams based on need.

**TABLE 9.** Similarities and Differences of iDARE Implementation in Uganda, Kenya, and Tanzania During the COVID-19 Pandemic

Step 1: Identify	
All	Involvement of local stakeholders to: Establish main goal using most recent available data. Determine priority gap(s) affecting the goal using most recent available data. Establish initial local iDARE team. Use iDARE journal to record team goal, indicator, and regularity of tracking progress.
Uganda	Used the 2020 USAID SBCA gender youth and social inclusion analysis to identify gaps in health outcomes based on social determinants, health, and geography as opposed to additional in-person focus group discussions and/or key informant interviews due to COVID-19 regulations. Reviewed with district local government DHIS2 data looking at all district progress to health goals – HIV (adherence and viral load suppression identified). Identified gaps and barriers experienced by nonsuppressed, actively enrolled clients through the formation of a small cohort using iDARE guide. Identified influencers of the cohort's men, children, and their caretakers to join iDARE team using iDARE guide. Analyzed information from cohort members using root cause analysis tool.
Kenya	Virtually reviewed program data on GBV service delivery performance and identified large GESI and service delivery gaps in GBV as opposed to in-person focus group discussions and/or key informant interviews due to COVID-19 regulations. Health workers submitted facility data as a baseline on GBV identification and management via email before training and informed iDARE training design as well as iDARE goals. Assessment also included input from the health workers on their biggest needs to improve GBV care in their health facility. Conducted desk review of national GBV service delivery tools, policies, guidelines, and standards, in comparison to international GBV standards. Identified capacity of health workers through a virtual assessment sent via email.
Tanzania	Used findings from the 2019 Act East GESI analysis as opposed to additional in-person focus group discussions and/or key informant interviews due to COVID-19 regulations. Virtual consultations with TZNTDCP staff on key socioecological factors impacting MDA coverage. Reviewed latest coverage evaluation surveys to identify patterns of groups missed during MDA to dig deeper. Identified gaps and barriers experienced by community members who had previously missed MDA through the formation of cohorts using iDARE guide. Identified influencers of the community members using iDARE guide. Analyzed information from community members using root cause analysis tool.
Step 2: Design	
All	Locally designed solutions to GESI identified gaps by iDARE team with support from iDARE coach. Expanded iDARE team to prepare for applying and assessing solutions. iDARE teams assigned roles and responsibilities to all team members. Used iDARE journal to log and track barriers and designed solutions to test.
Uganda	Virtual iDARE training on GESI concepts with supervisors and iDARE teams. Short, high-frequency sessions and telecoaching designed to support iDARE implementation. In-person coaching with team leader if restrictions did not allow for full group. Hybrid and purely virtual coaching when interdistrict travel was not allowed. Expanded iDARE teams included community influencers, identified by actively enrolled male and children clients, using semistructured interviews with nonsuppressed clients (using iDARE guide).
Kenya	Virtual GBV training designed to be staggered over 6 weeks, intentionally designed to be mindful of re-traumatization and do no harm. Virtual training designed as low dose, high frequency with practical “homework” for health workers to utilize skills learned in virtual training. Training content designed based on the capacity assessment taken by health workers in advance. iDARE training sessions designed based on the health worker capacity assessment and baseline data submitted by facility teams. Three health workers specifically selected for each facility by county government supervisors based on their role in GBV identification, management, and response. Additional team members were added to iDARE team after the training. Connected all training participants via WhatsApp groups to share experiences and solve issues together.
Tanzania	Mixed virtual and in-person national training of trainers (TZNTDCP staff). Virtual collaboration and development with national trainers on GESI tools and materials for national trainings. Revised all in-person trainings to have multiple rooms (in same location, so minimal participants in a room) connected virtually. Team members established at start of implementation.
Step 3: Apply and Assess	
All	Regular iDARE team meetings with coaching support to: Review progress against set indicator. Establish if solutions are successful or not successful and next steps. Design new solutions to existing and or new gaps identified.
Uganda	Established learning session between Kenya and Uganda iDARE coaches to share experiences in supporting iDARE implementation during COVID-19 lockdowns and restrictions.
Kenya	Established learning session between Kenya and Uganda iDARE coaches to share experiences in supporting iDARE implementation during COVID-19 lockdowns and restrictions. Incorporated feedback into every day of training (logistics, content, etc.) to adapt and revise the next day's content and plan. Used facility data on GBV identification and management to inform progress of health workers' application of new skills and knowledge gained throughout trainings.
Tanzania	Rapidly adapted planned 4-month behavior change work plan to be completed in 3 weeks once approval was granted. Revised all meeting and informal interview (using iDARE guide) plans due to regulations in place on meeting sizes. Formed multiple smaller cohorts to conduct informal interviews.
Step 4: Record	
All	Progress on iDARE journals recorded by iDARE team lead. Qualitative and quantitative data regularly captured by iDARE team and coach. Training and coaching reports by iDARE coach. Data dashboards developed for iDARE coach to see progress. Pre- and post-capacity assessments recorded to measure progress of trainings and capacity-building sessions.
Kenya	Conducting study on impact of iDARE during COVID-19.
Step 5: Expand	
All	Expansion of learnings through: Internal and external presentations – for example, shared results during conference sessions, including GHTechX. Webinars and blogs.
Uganda	Learning sessions between Kenya and Uganda iDARE coaches to share and expand learnings. Expanded iDARE solutions beyond initial cohort to all active in care male and children clients. Both facilities expanded iDARE work to additional issue areas, TB case notification, and lost to follow-up.
Kenya	Learning sessions between Kenya and Uganda iDARE coaches to share and expand learnings. iDARE teams worked on expanding their efforts in the community to ensure that individuals who have been subjected to violence know there are support services available to them at the facility.
Tanzania	Applied lessons learned and adapted tools to Uganda under NTD work (and currently adapting for Nepal). Use the lessons learned for expansion to 6 additional councils in Tanzania.

Abbreviations: COVID-19, coronavirus disease; DHIS, district health information system; GBV, gender-based violence; GESI, gender equity and social inclusion; GHTechX, Global Health Tech Exchange; MDA, mass drug administration, NTD, neglected tropical disease; SBCA, Social and Behavior Change Activity; TZNTDCP, Tanzania National NTD Control Program; USAID, U.S. Agency for International Development.

In all 3 countries, iDARE coaches supported the iDARE teams to identify barriers and gaps to address, while challenging their own internal conscious or unconscious biases as well as their assumptions in relation to their goal. In Uganda and Tanzania, this meant challenging their perceived notions and ideas of why men and children were not able to achieve VLS and why school children were missing during MDA, respectively. In Kenya, health workers challenged their biases regarding who can be subjected to violence and what a survivor looks like. The iDARE team coach trained the iDARE team members on GESI concepts, including gender and power dynamics, stigma, and bias, and went through practical exercises to identify and understand subconscious and conscious bias in their lives, beyond just the health area of focus. Through the interviews with the target populations themselves, teams learned about unexpected influencers in their clients' lives, as well as GESI and other barriers, that they were unaware were affecting health seeking and utilization behavior.

A critical component of forming iDARE teams is ensuring inclusive representation of the various stakeholders, with an emphasis on the importance of including community-selected influencers. By bringing together these individuals to achieve a goal to transform their community, iDARE teams can recognize, challenge, and even in some cases, leverage existing power dynamics to stimulate change for their community. iDARE teams continuously interact with the target population, most often through the influencers, to ensure their voices, perspectives, and beliefs are heard throughout implementation. The iDARE coach emphasizes the importance of prioritizing groups that are often excluded or missing to ensure inclusive and appropriate design and delivery of solutions to change community outcomes. Furthermore, the iDARE team coach provides ongoing capacity building to teams to ensure adherence to the “do no harm” principle.

A critical component of forming iDARE teams is ensuring inclusive representation of the various stakeholders, with an emphasis on including community-selected influencers.

### Ethics Approval

Ethical considerations, including informed consent among cohorts and iDARE teams, were adhered to throughout implementation. Individuals in the participating cohorts and in relevant cases, their caretakers, gave both verbal and written consent or assent. Informal interviews conducted were never recorded. No identifying information was ever captured for data collection or analysis.

### Limitations

Limitations exist for the implementation of the iDARE methodology. First, to ensure effective application of the methodology, including the identification of GESI gaps, barriers, and issues affecting an outcome, as well as influencing factors to design culturally appropriate solutions, individuals must be adequately sensitized to GESI and the principle of do no harm. Furthermore, application and adherence to the do no harm principle takes practice and patience to develop the skills and understanding over time. Lastly, data are critical to the implementation of iDARE, and the methodology is to be applied within an existing system, which means the use of existing data—usually government data. Data are not always regularly available or reliable.^15^ WI-HER works directly with all implementers of iDARE to acknowledge and establish early solutions to avoid these limitations from the start through sensitizations, trainings, and in some cases the application of data process improvement efforts.

We also acknowledge the limitations of the results presented in this field action report. The results we present are program implementation results, and no comparative or control cases were factored into this work. Additional investigations are needed to explore the longitudinal impact of iDARE as well as the complexity of relationships between the wide range of actors at the different levels of implementation. Additional qualitative and quantitative data are recommended to explore shifts over time in gender and social norms and behaviors as well as in complex power relations while using iDARE.

## CONCLUSION

iDARE leveraged local-led transformation that produced community-based change in 3 separate contexts. It incited codesigning of culturally sensitive, relevant SBC approaches, including appropriate measures that reflected the lived experiences of the target population, while addressing data collection and usage constraints across all levels.^16^ iDARE teams must always consist of the relevant local stakeholders, with particular attention to inclusivity and representation of all actors within the socioecological environment.

Each country used the methodology to guide them, with less-than-intended coaching support due to COVID-19 restrictions, through critical thinking, local solutions, using existing resources, and navigating all challenges, especially those arising during the COVID-19 pandemic. Results from the 3 countries during the pandemic show that iDARE is collaborative, empowering, and impactful in achieving improved health outcomes through behavior change and/or system improvement efforts. The key principles and adaptability of iDARE are applicable beyond the pandemic setting, as the methodology allows for local actors to adapt and contextualize the methodology to any setting, using existing resources, to achieve an intended goal. Further studies on the use of iDARE in various contexts are recommended to gain additional insight into the methodology's usage to shift power, gender, and social norms as well as overall SBC.

## Notes

**Peer Reviewed**
